# Supplementary material for: Transposable Elements are Dysregulated in Brains of Individuals with Major Depressive Disorder
Source: bioRxiv. 2025 Jan 24:2025.01.22.634143. Preprint. [Version 1] doi: 10.1101/2025.01.22.634143 (PMC11785094; doi:10.1101/2025.01.22.634143)
Supplement: 1 [file NIHPP2025.01.22.634143v1-supplement-1.pdf]

bioRxiv preprint doi: https://doi.org/10.1101/2025.01.22.634143; this version posted January 25, 2025. The copyright holder for this preprint (which was not certified by peer review) is the author/funder, who has granted bioRxiv a license to display the preprint in perpetuity. It is made available under aCC-BY 4.0 International license.

Supplemental Information (SI) Appendix

Extended Materials and Methods:

We conducted a comprehensive analysis of publicly available RNAseq data from human postmortem brain tissue[23] across six brain regions: the orbitofrontal cortex (OFC; BA11), dorsolateral prefrontal cortex (dlPFC; BA8/9), ventromedial prefrontal cortex (vmPFC; BA25), anterior insula (aINS), nucleus accumbens (NAc), and ventral subiculum (vSUB). Our annotation of TEs was performed using TETranscripts[55] allowing us to generate a list of differentially expressed TEs (DETEs), as well as differentially expressed genes (DEGs), in individuals diagnosed with MDD compared to matched healthy controls (CTRLs). The analysis included a total of 26 postmortem samples from MDD subjects (comprising 13 males and 13 females) and 22 control samples (13 males; 9 females), and used a model that considers all covariates (including sex, age, medication, smoking).

RNA-seq data preprocessing and quality control

Publicly available RNAseq datasets were downloaded from NCBI GEO GSE102556. Raw RNA-seq FASTQ files were subjected to quality control using FastQC (version 0.11.9) to assess read quality. Adapter sequences were removed using Trimmomatic (version 0.39) with further trimming off low-quality bases (10 bases from the head, and 3 base from the tail, respectively). In addition, we used a sliding window to trim off the bases with average base quality lower than 20. Reads shorter than 16 (half of 50-10-3) were also be dropped.

RNAseq read alignment and TE/gene quantification

After the quality control, high-quality reads were aligned to the Homo sapiens GRCh38 reference genome for human data available on Ensembl using STAR (version 2.7.11a) with the recommended parameters. We employed TEtranscripts (version 1.09)[55] in the quantification of both gene and TE expression levels by integrating genomic and RepeatMasker annotations (Hammell lab).

Differential Expression Analysis

The RNAseq count data were normalized, and dispersion estimates were obtained according to DESeq2's standard pipeline. We employed a model in which age, sex, medication and smoking history were adjusted as covariates. Medication type was categorized into Alzheimer's disease, anti-convulsant, antipsychotic, lithium, and no medication. Smoking history was categorized into heavy,

bioRxiv preprint doi: https://doi.org/10.1101/2025.01.22.634143; this version posted January 25, 2025. The copyright holder for this preprint (which was not certified by peer review) is the author/funder, who has granted bioRxiv a license to display the preprint in perpetuity. It is made available under aCC-BY 4.0 International license.

moderate, and non-smoker. When comparing MDD vs CTRL samples, and in looking at sex and age, our DE analysis model utilized the whole-brain counts with all brain region samples combined. The RNAseq counts from different brain regions of the same subject were averaged to make the (pseudo-) whole-brain counts. Wald tests were performed to determine the significant levels of differentially expressed genes (DEGs) or TEs (DETEs) between the two clinical conditions (MDD vs. CTRL). The DEGs and DETEs were considered significant if the absolute log2 fold change were greater than 1 and the false discovery rate (FDR) less than 0.05. Volcano plots with these DEGs and DETEs were assembled in GraphPad Prism 10.

Genomic annotation of TEs and potential regulated genes

We annotated the TEs of overlapping genomic features. The genomic features included in this analysis were the promoter regions, enhancers, histone H3K4 tri-methylation sites and CTCF binding sites revealed by ENCODE project, as well as the exon, intron, UTR from GRCh38 gene annotations. The overlapping genomic features were identified based on a minimum of 1 bp overlap of the chromosomal position between the observed TEs and the genomic features. The genes with introns overlapping with TEs were identified directly from gene annotations. The genes that are potentially regulated by the overlapping enhancers were revealed by analyzing the data of various high-throughput experiments, e.g. histone modifications, DNase-seq, or ChIA-PET. Downregulated DEGs in MDD vs CTRL conditions ($p < 0.05$ cutoff) that harbor intronic TEs were analyzed using SRplot gene ontology generator[56].
